# Mitochondrial Sirtuin TcSir2rp3 Affects TcSODA Activity and Oxidative Stress Response in *Trypanosoma cruzi*

**DOI:** 10.3389/fcimb.2021.773410

**Published:** 2021-11-11

**Authors:** Leila dos Santos Moura, Vinícius Santana Nunes, Antoniel A. S. Gomes, Ana Caroline de Castro Nascimento Sousa, Marcos R. M. Fontes, Sergio Schenkman, Nilmar Silvio Moretti

**Affiliations:** ^1^ Laboratório de Biologia Molecular de Patógenos, Escola Paulista de Medicina, Universidade Federal de São Paulo, São Paulo, Brazil; ^2^ Departamento de Microbiologia, Imunologia e Parasitologia, Escola Paulista de Medicina, Universidade Federal de São Paulo, São Paulo, Brazil; ^3^ Departamento de Biofísica e Farmacologia, Instituto de Biociências, Universidade Estadual Paulista (UNESP), Botucatu, Brazil

**Keywords:** *Trypanosoma cruzi*, acetylation, superoxide dismutase, sirtuins, oxidative stress

## Abstract

*Trypanosoma cruzi* faces a variety of environmental scenarios during its life cycle, which include changes in the redox environment that requires a fine regulation of a complex antioxidant arsenal of enzymes. Reversible posttranslational modifications, as lysine acetylation, are a fast and economical way for cells to react to environmental conditions. Recently, we found that the main antioxidant enzymes, including the mitochondrial superoxide dismutase A (TcSODA) are acetylated *in T. cruzi*, suggesting that protein acetylation could participate in the oxidative stress response in *T. cruzi*. Therefore, we investigated whether mitochondrial lysine deacetylase TcSir2rp3 was involved in the activity control of TcSODA. We observed an increased resistance to hydrogen peroxide and menadione in parasites overexpressing TcSir2rp3. Increased resistance was also found for benznidazole and nifurtimox, known to induce reactive oxidative and nitrosactive species in the parasite, associated to that a reduction in the ROS levels was observed. To better understand the way TcSir2rp3 could contributes to oxidative stress response, we analyzed the expression of TcSODA in the TcSir2rp3 overexpressing parasites and did not detect any increase in protein levels of this enzyme. However, we found that these parasites presented higher levels of superoxide dismutase activity, and also that TcSir2rp3 and TcSODA interacts *in vivo*. Knowing that TcSODA is acetylated at lysine residues K44 and K97, and that K97 is located at a similar region in the protein structure as K68 in human manganese superoxide dismutase (MnSOD), responsible for regulating MnSOD activity, we generated mutated versions of TcSODA at K44 and K97 and found that replacing K97 by glutamine, which mimics an acetylated lysine, negatively affects the enzyme activity *in vitro*. By using molecular dynamics approaches, we revealed that acetylation of K97 induces specific conformational changes in TcSODA with respect to hydrogen-bonding pattern to neighbor residues, suggesting a key participation of this residue to modulate the affinity to ${\text{O}}_{2}^{-}$ . Taken together, our results showed for the first time the involvement of lysine acetylation in the maintenance of homeostatic redox state in trypanosomatids, contributing to the understanding of mechanisms used by *T. cruzi* to progress during the infection.

## Introduction

*Trypanosoma cruzi* is a protozoan parasite of the Trypanosomatidae family responsible for Chagas disease, an illness endemic in 21 countries across Latin America and now being detected in the southern USA and many other countries. Around 7 million people are infected and more than 70 million live at risk to be infected in the world (Perez-Molina and Molina, 2018; Lidani et al., 2019; Moretti et al., 2020). The parasite has a heteroxenous life cycle shifting between an invertebrate and a vertebrate host. Metacyclic trypomastigote parasites are transmitted by triatomine bug feces during blood meal and can infect any nucleated cell type. Inside the vertebrate host cell, the metacyclic trypomastigotes differentiate to amastigote replicative forms, which latter on will differentiate to trypomastigotes that will be released upon cell lyses and can infect other cells or be ingested by triatomine bugs during a blood meal. In the invertebrate host, trypomastigotes differentiate to replicative epimastigotes that in the hindgut will generate metacyclic trypomastigotes, which can infect another vertebrate host (Perez-Molina and Molina, 2018; Moretti et al., 2020). During its life cycle transitions, *T. cruzi* faces variable environmental conditions, such as, changes in temperature, nutritional state, and oxidant products, and the parasite must adapt to these alterations to survive and succeed in the infection (Moretti and Schenkman, 2013).

Reversible posttranslational modifications (PTMs) are a fast and economical way for cells to react to environmental conditions. PTMs such as methylation, phosphorylation, and acetylation are detected on hundreds of proteins in the cells (Choudhary et al., 2014; Levy, 2019; Macek et al., 2019). Acetylation of lysine residues is one of the most common PTMs and is characterized by the addition of an acetyl group to the ε-amino group of this protein residue. The acetyl group neutralizes the lysine positive charge conferring novel properties to the modified proteins, from changes in enzymatic activity to subcellular localization (Narita et al., 2019).

This PTM has been identified in several prokaryote and eukaryote species (Carabetta and Cristea, 2017; Narita et al., 2019; Maran et al., 2021). Recently, we have described the acetylome, the set of lysine-acetylated proteins, of procyclic and bloodstream forms of *T. brucei* and of *T. cruzi* epimastigotes (Moretti et al., 2018). We detected 288 lysine acetylation sites (Kac) in 210 proteins of procyclic form and 380 Kac in 285 proteins in the bloodstream form, and in *T. cruzi* epimastigotes, we found 389 Kac sites in 235 proteins (Moretti et al., 2018). Interestingly, in *T. brucei*, glycolytic enzymes were found to be heavily acetylated and later proved to be regulated by acetylation (Barbosa Leite et al., 2020), while in *T. cruzi*, several proteins that are central for the antioxidant defense of the parasite were detected to be acetylated (Moretti et al., 2018).

The addition and removal of acetyl groups on lysines are coordinated by lysine acetyltransferases (KATs) and lysine deacetylases (KDACs), respectively (Marmorstein and Zhou, 2014; Seto and Yoshida, 2014). The KDACs are subdivided into four classes (I, II, III/sirtuins, IV). Class III KDACs, also known as sirtuins, are homologous to yeast Sir2 and require nicotinamide adenine dinucleotide (NAD^+^) as a cofactor for their catalytic activity (Seto and Yoshida, 2014). Sirtuins are conserved from bacteria to humans (Brachmann et al., 1995). These are multifunctional enzymes involved in several biological processes, as gene expression regulation, DNA repair, metabolism, and oxidative stress response (Choi and Mostoslavsky, 2014). The number of genes coding for sirtuins varies depending on the species; while humans have seven sirtuins, bacteria have only one. Three sirtuins are present in *T. brucei* (TbSir2rp1-3) and *Leishmania* spp. (LmSir2rp1–3), while only two are described in *T. cruzi* (TcSir2rp1 and 2) (Alsford et al., 2007; Tavares et al., 2008; Moretti et al., 2015; Ritagliati et al., 2015; Vergnes et al., 2016).

The TcSir2rp1 and TcSir2rp3 sirtuins have cytosolic and mitochondrial localization, respectively (Moretti et al., 2015; Ritagliati et al., 2015). Overexpression of TcSir2rp3 improves epimastigote multiplication and epimastigote differentiation to infective metacyclic forms. Moreover, overexpression increases amastigote intracellular multiplication, a phenotype that is negatively affected when an inactive version of TcSir2rp3 was overexpressed or specific sirtuin inhibitors, such as salermide, are used (Moretti et al., 2015).

In this work, we investigated the involvement of *T. cruzi* mitochondrial sirtuin in the regulation of oxidative stress response of the parasite. We investigated whether overexpression of TcSir2rp3 increases parasite resistance to oxidative stress and if the mitochondrial superoxide dismutase A could be regulated by TcSir2rp3. We further verified the enzymatic activity of recombinant forms of TcSODA to investigate the effects of acetylation and looked by molecular dynamic analysis the predicted effects of acetylation on the enzyme activity. Our results indicate for the first time a direct effect of lysine acetylation in the regulation of antioxidant enzymes in protozoan parasites.

## Materials and Methods

### Cloning of Native and Mutated Versions of the TcSODA protein

To obtain the native version of *T. cruzi* SODA protein (TcCLB.509775.40) (http://tritrypdb.org), the gene was PCR amplified from genomic DNA of *T. cruzi* Y strain using the oligonucleotides TcSODA-Fow (5′-TCTAGACATATGTTGAGACGTGCGGTGAA-3′), TcSODA-Rev (5′-CCGGATCCTTATTTTATGCCTGCGCATGCAT-3′). The underlined residues represent cloning sites, for *Nde*I and *Bam*HI enzymes, respectively. To generate mutated TcSODA proteins, we used two rounds of PCRs. In the first round, for TcSODA-K44Q and TcSODA-K97Q, where the lysine (K) residues were replaced by glutamine (Q), by changes in the nucleotides A130 and A289 to cytosines, respectively. For TcSODA-K97R, the K was substituted by arginine (R) replacing the nucleotide A289 by guanine. In the second round of PCR, we used the same primers described above to obtain the native version of TcSODA. The oligonucleotides used to obtain the mutated TcSODA versions are listed in **Supplementary Table S1**. The native and mutated TcSODA fragments were digested with *Nde*I and *Bam*HI and cloned into the expression vector pET28a (New England Biolabs, Ipswich, MA, USA), using the same restriction sites. Clones were selected and submitted to sequencing reactions to confirm the desired mutations.

### TcSODA Protein Heterologous Expression and Purification

The pET28a vectors bearing native or mutated versions of TcSODA (K44Q, K97R, and K97Q) were transformed into BL21 bacteria (DE3) (New England Biolabs); clones were selected and used for protein heterologous expression. For the expression, bacteria were grown at 37°C in LB medium containing 50 µg/ml kanamycin and induced by addition of 0.1 mM IPTG at 0.6–0.8 OD at absorbance of 600 nm and incubated for an additional 4 h. The cells were collected by centrifugation at 5,000×*g* for 10 min, washed once with 20 mM Tris-HCl, pH 8.0, and the pellets were resuspended in lysis buffer (200 mM NaCl, 5% glycerol, 5 mM 2-mercaptoethanol, 25 mM HEPES-NaOH, pH 7.5, and proteases inhibitors) and lysed using the French Press apparatus to obtain the soluble fractions of proteins. The soluble fractions were incubated with Ni-NTA Agarose resin (QIAGEN, Hilden, Germany), previously equilibrated with equilibrium buffer (200 mM NaCl, 5% glycerol, 5 mM 2-β-mercaptoethanol, 25 mM imidazole, 25 mM HEPES-NaOH, pH 7.5). After 30 min under agitation at 4°C, the resin was washed four times with 5 ml of wash buffer (200 mM NaCl, 5% glycerol, 5 mM 2-β-mercaptoethanol, 50 mM imidazole, 25 mM HEPES-NaOH, pH 7.5) and proteins were eluted with 4 ml of elution buffer (200 mM NaCl, 5% glycerol, 5 mM 2-β-mercaptoethanol, 250 mM imidazole, and 25 mM HEPES-NaOH, pH 7.5). The quality of the purification was confirmed by SDS-PAGE gel electrophoresis and staining with Coomassie blue for further use.

### TcSODA Enzyme Activity Assays

The activity of superoxide dismutase was determined using the oxidation of 4-Nitro blue tetrazolium chloride (NBT) (Sigma-Aldrich, St. Louis, MO, USA) method, as described in Ewing and Janero (1995). To measure the activity of purified recombinant TcSODA proteins (native and mutated versions), proteins were initially dialyzed against the superoxide dismutase reaction buffer (50 mM KPO_4_, 0.1 M EDTA, pH 7.4), concentrated using Amicon Ultra-10 K columns (Merck Millipore, Burlington, MA, USA) and quantified with Pierce™ BCA Protein Assay Kit (Thermo Fischer Scientific). A total of 10 µg of dialyzed proteins (native and mutant versions) were incubated with 1× reaction buffer (50 mM KPO_4_, 0.1 M EDTA, pH 7.4), 50 µM NBT (Sigma-Aldrich), 78 µM NADH (Sigma-Aldrich), and 3.3 µM of phenazine (Sigma-Aldrich), in a 96-well plate. The enzymatic activity was determined by the detection of NBT oxidation at absorbance of 560 nm using the SpectraMax M3 equipment (Molecular Devices, San Jose, CA, USA), every 30 s during 5 min. When indicated, 5 µg of purified TcSODA was incubated in 50 mM Tris-HCl at pH 7.4 with different concentrations of anhydride acetic at 1, 2.5, 5, and 10 µM at room temperature for 30 min, and the samples were dialyzed against 50 mM Tris-HCl at pH 7.4.

The superoxide dismutase activity in *T. cruzi* extracts of wild-type, Sir2rp3-WT, and Sir2rp3-MUT parasites, previously described in Moretti et al. (2015), was performed using epimastigote forms at exponentially growing phase. The parasites were collected by centrifugation and washed in PBS 1× and total protein extracts obtained by freezing and thaw in PBS 1× and 1× Complete-EDTA free protease inhibitors and submitted to dialysis against SOD reaction buffer (50 mM KPO_4_, 0.1 M EDTA, pH 7.4) and further used to activity assays as described above.

### Parasite Cultivation and Growth Curve Experiments

For this work, we used *T. cruzi* Y strain wild type and two cell lines overexpressing the mitochondrial sirtuin, TcSir2rp3, in its native (Sir2rp3-WT) and inactive (Sir2rp3-MUT) forms, generated previously (Moretti et al., 2015). Epimastigote forms of all cell lines were cultivated in liver infusion tryptose (LIT) medium supplemented with 10% fetal bovine serum (FBS) at 28°C, and for transfectant parasites, 500 µg/ml of Geneticin (G418) was added to the medium.

The effect of hydrogen peroxide (H_2_O_2_) was determined in epimastigotes inoculated at 2 × 10^7^ parasites/ml in LIT medium containing 100 µM of H_2_O_2_ at 28°C. After 20 min, the parasite cells were collected by centrifugation and resuspended in fresh LIT medium without H_2_O_2_, and parasite numbers was measured after 24 h with Neubauer chamber. In the experiments with menadione, 5 × 10^6^ parasites/ml epimastigote forms of all cell lines were inoculated in LIT medium in the presence of 10 µM of the drug, and the parasite growth was determined after 48 h. The experiments using nicotinamide were carried out as described above, but with the addition of 10 mM of nicotinamide in the described conditions.

To determine the resistance of cell lines against benznidazole (BZD) and nifurtimox (NFX), 2 × 10^6^ parasites/ml epimastigote forms were inoculated in LIT medium in 24-well plates, in the presence of increasing drug concentrations and kept growing at 28°C for 4 days. After the incubation time, parasite cell growth was determined by counting the cells with Neubauer chamber, and BZD/NFX resistance was calculated by comparing parasite growth in the absence versus parasite growth in the presence of drugs. The experiments in the presence of salermide were performed as described above, with addition of 25 µM of the inhibitor.

### Quantification of Reactive Oxygen Species Levels

To determine the levels of reactive oxygen species (ROS), the parasites were initially exposed to 25 μg/ml of BZD or 15 μM of NFX for 24 h in LIT medium at 28°C. These concentrations correspond to twice the EC_50_ of these drugs in the employed *T. cruzi* lineage previously determined. After incubation, 1.5 × 10^7^ parasites were collected from each cell line and incubated for 30 min with 5 μM of CellROX Deep Red (#C10422, Thermo Fisher Scientific, Waltham, MA, USA) or CellROX Green (#C10444, Thermo Fisher Scientific) reagents, to measure the cytoplasmic and mitochondrial ROS, respectively. The parasites were then washed twice with PBS 1×, and the fluorescence was measured using flow cytometry with BD Accuri™ C6 Flow Cytometer (BD Biosciences, Franklin Lakes, NJ, USA) equipment. As a positive control of the experiment, parasites were exposed to 100 μM H_2_O_2_ for 20 min and washed once with PBS 1× before incubation with both reagents. All experiments were carried out in triplicate.

### Protein Immunoprecipitation Assays

Approximately 1 × 10^9^ cells were collected, washed once in PBS, and used to obtain total protein extracts of exponential cultures (1–1.5 × 10^7^ cells/ml) of the epimastigotes grown in LIT medium at 28°C. The parasites were lysed in lysis buffer (20 mM Tris-HCl, pH 8.0; 137 mM NaCl; 2 mM EDTA; 1% Triton X-100; 25 mM nicotinamide; 25 mM sodium butyrate and protease inhibitors), and the soluble fraction was collected after centrifugation for 10 min, at 13,000×*g* at 4°C. The soluble fraction was incubated with Dynabeads Protein A (Thermo Fisher Scientific), previously coupled with anti-TcSODA or anti-TcSir2rp3 antibodies, obtained during this work in our laboratory. After 2 h of incubation, samples were washed three times with washing solution (10 mM Tris-HCl pH 7.4; 1 mM EDTA; 1 mM EGTA; 150 mM NaCl; 1% Triton X-100; and 1× Complete-EDTA free protease inhibitors), followed by elution of the immunoprecipitated proteins with 0.2 M of glycine at pH 2.0. The samples were then neutralized and used in the Western blot experiments to detect the protein interactions with specific antibodies.

### Western Blot Experiments

Protein extracts were obtained by lysing the parasites with lysis buffer (PBS 1×, 1% of Triton X-100, 0.5 mM of phenyl-methylsulfonyl fluoride (PMSF), and 1× Complete-EDTA free protease inhibitors. The samples were mixed with 2× Laemmli Buffer (Sigma-Aldrich) and used in SDS-PAGE electrophoresis with 12.5% polyacrylamide gels. After electrophoresis, the proteins were transferred to nitrocellulose membranes (Bio-Rad Laboratories, Hercules, CA, USA) using the Trans-blot SD Semi-Dry system (Bio-Rad Laboratories). The membranes were incubated with a blocking solution (1× PBS, 0.01% Tween 20, and 5% skimmed milk powder) for 1 h under agitation, followed by incubation with specific primary antibodies diluted in blocking solution, anti-TcSir2rp3 (1:2,500), anti-TcSODA (1:5,000), anti-Histone H3 (1:10,000), anti-aldolase (1:5,000), and antiacetyl-lysine (1:2,000) for 1 h under agitation at room temperature. The membranes were washed three times with PBS 1× containing 0.01% Tween 20 and incubated with secondary antibodies IRDye^®^ 800CW Goat anti-Rabbit IgG Secondary Antibody or IRDye^®^ 700CW Goat anti-Mouse IgG Secondary Antibody diluted 1:10,000 in blocking buffer (Li-Cor, Lincoln, NE, USA), during 1 h at room temperature. The membranes were washed three times with washing solution and revealed using Odyssey Li-Cor equipment. The same procedure was used for Western blot analysis with samples obtained in protein immunoprecipitation assays or for samples from acetic anhydrite assays.

The primary antibodies anti-TcSODA and anti-TcSir2rp3 were house made during this work; anti-aldolase was obtained previously in our laboratory (Barbosa Leite et al., 2020), anti-histone H3 was kindly provided by Prof. Dr. Christian Janzen from the University of Würzburg, Germany, and antiacetyl-lysine was purchased from Millipore (#ab80178).

### Molecular Dynamics Simulations of TcSODA

The crystallographic structure of TcSODA was retrieved from the Protein Data Bank (PDB ID: 4H3E) (Phan et al., 2015), preserving only the coordinates of Fe, and submitted to molecular dynamics (MD) simulations using GROMACS v.2020.6 under the CHARMM36m force field (Huang et al., 2017). The protonation state of TcSODA was determined according to the PROPKA3 webserver (Olsson et al., 2011). To study the effect of the acetylation in K97, TcSODA was used as a monomer in both native (WT) and acetylated (K97Ace) states. Initially, each configuration of TcSODA was centered in a rhombic dodecahedric box of 12 Å distant from the farthest atom in XYZ directions. Furthermore, each system was solvated and equilibrated with 0.1 M of NaCl, with some additional ions added to reach a system with zero charge. As previously described, iron superoxide dismutases show a Fe^3+^ bound to the protein at the first step of the enzymatic process, thus, the Fe^3+^ topology was obtained from a previous work in order to reproduce the same conformational state of the enzyme (Li et al., 2015). Each system was minimized using the Steepest Descent algorithm until reaching an energy gradient below 100 kJ/mol/nm^2^. As follows, each system was submitted to an NVT ensemble generating the initial velocities randomly following a Maxwell-Boltzman distribution at 300 K during 1 ns, monitoring the temperature using the V-Rescale thermostat (Bussi et al., 2007) with a time constant of 0.1 ps. An NPT ensemble of 1 ns was then applied maintaining the pressure of the system at 1 bar using the Berendsen barostat (Eslami et al., 2010). Both steps were performed restraining the backbone atoms of the protein and Fe^3+^ with a force constant of 1,000 kJ/mol/nm^2^. Finally, an unconstrained MD of 200 ns was applied using the Nose-Hoover thermostat (Hoover, 1985) and Parrinello-Rahman barostat. Nonbonded interactions were calculated considering atoms within 10 Å using the PME method, with a switching force function between 10 and 12 Å. Three independent replicas for both wild-type and acetylated systems were performed collecting frames every 10 ps.

Two structural analyses were performed to study the acetylation effect on TcSODA: (i) the volume occupancy of the sidechain nitrogen atom of K97 or K97Ace was calculated using the VolMap tool at a resolution of 1 Å and Isovalue of 0.25; and (ii) the minimal distance of the sidechain nitrogen atom of K97 to the sidechain oxygen atoms of D94 or E96 residues of TcSODA. Both analyses were performed using VMD (Humphrey et al., 1996).

### Ethics Committee

This work was carried out in accordance with the rules of the Research Ethics Committee (CEP) of the Universidade Federal de São Paulo (UNIFESP). The experimental protocol was approved by the CEP under number 6383311017.

## Results

### Mitochondrial *T. cruzi* Sirtuin Overexpression Increases Parasite Resistance to H_2_O_2_ and Menadione

As sirtuins regulate the levels of protein acetylation, and in *T. cruzi*, several proteins involved in redox reactions are acetylated (Moretti et al., 2015), we initially explored how TcSir2rp3 could impact parasite responses to oxidative stress. For this, we employed the *T. cruzi* cell lines overexpressing the mitochondrial sirtuin, TcSir2rp3, in its native (Sir2rp3-WT) and inactive versions (Sir2rp3-MUT). These two cell lines expressed similar levels of TcSir2rp3 which were about three times than the endogenous protein in the nontransfected line, as demonstrated in Moretti et al. (2015).

**Figure 1:**
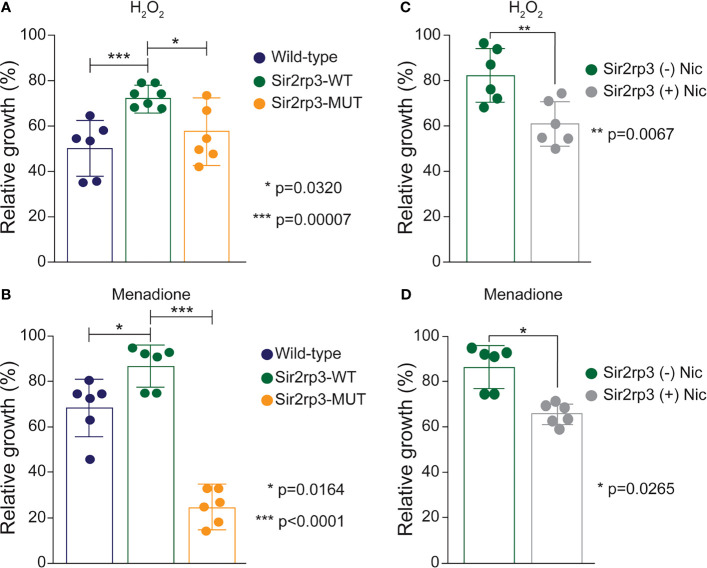
Overexpression of mitochondrial TcSir2rp3 improves parasite recovery after oxidative stress. **(A, B)** Epimastigote forms of WT (blue bars), Sir2rp3-WT (green bars), and Sir2rp3-MUT (orange bars) were submitted to oxidative stress with 100 µM H_2_O_2_ and 10 µM menadione and recovering was quantified. **(C, D)** Sir2rp3-WT (green bars) recovery was analyzed in the assays performed in the presence of nicotinamide (Nic) (grey bars) compared with assays performed without the inhibitor (orange bars). All experiments were performed in triplicate. Statistical analyzes were performed using the Student’s *t*-test. ^*^ *p* = 0.0320; ^***^ *p* = 0.00007 in **(A)**; ^**^ *p* = 0.0067 in **(B)**; ^*^ *p* = 0.00164 and ^***^ *p* < 0.0001 in **(C)**; ^*^ *p* = 0.0265 in **(D)**.

The different epimastigote cell lines were subjected to oxidative stress by hydrogen peroxide (H_2_O_2_) and menadione, and the recovering after stress was determined. In addition to a faster growth rate (Moretti et al., 2015), the Sir2rp3-WT cell line recovered faster from the oxidative stress caused by H_2_O_2_ compared with WT or Sir2rp3-MUT parasites (**Figure 1A**). In the case of menadione, a better recovery was detected for Sir2rp3-WT, but a large reduction in parasite growth was observed in Sir2rp3-MUT parasites compared with WT cell line (**Figure 1C**).

To investigate if the phenotype observed for Sir2rp3-WT overexpressor was directly related to enzyme activity, we performed the same assays in the presence of nicotinamide, a sirtuin inhibitor. A reduction in the recovering phenotype was detected in the presence of nicotinamide in the Sir2rp3-WT cells, compared with those cultivated in the absence of nicotinamide (**Figures 1B, D**).

### TcSir2rp3 Overexpression Increases Parasite Resistance to BZD and NFX

Based on the findings that TcSir2rp3 improves *T. cruzi* recovering after H_2_O_2_ and menadione stress and in the fact that the two drugs available for treatment of Chagas disease, nifurtimox (NFX) and benznidazole (BZD), acts through oxidative damaged on the parasites, we decided to evaluate the effect of TcSir2rp3 overexpression in BZD and NFX resistance.

**Figure 2:**
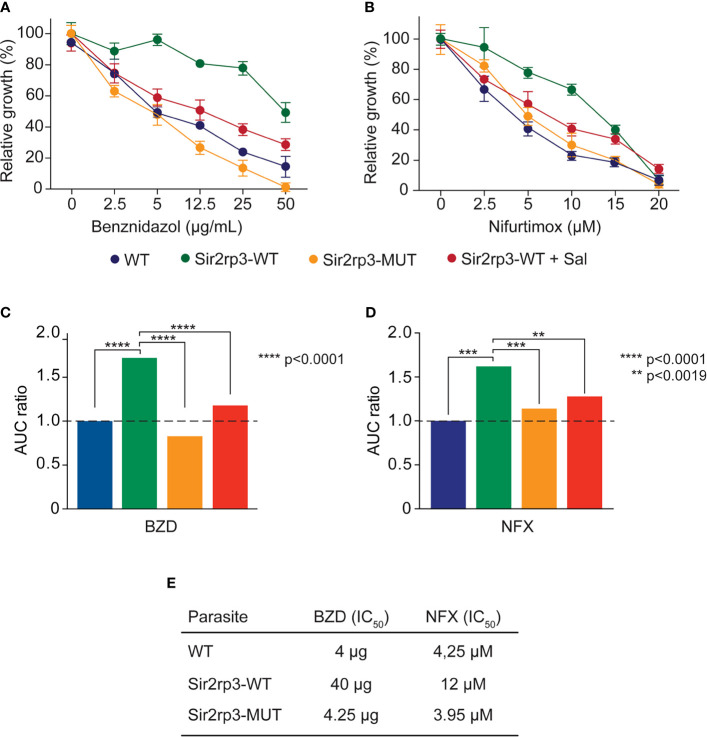
TcSir2rp3 increases parasite resistance to BZD and NFX. Epimastigotes of the indicated lines were incubated with BZD **(A, B)** or NFX **(C, D)** and the relative growth determined by the percentage of parasites in the presence or absence of each compound. All the values are mean ± SD of triplicates. To better visualize the effects, **(B, D)** show the area under the curve (AUC) of the data shown in **(A, C)**. **(E)** IC_50_ of BZD and NFX of the WT, Sir2rp3-WT, and Sir3rp3-MUT cell lines. The IC_50_ values were calculated based on the growth curve obtained in **(A, B)**.

For that, epimastigotes of WT, Sir2rp3-WT, and Sir2rp3-MUT were cultivated in the presence of increasing concentrations of BZD and NFX, and their survival was evaluated after 4 days of growing compared with mock-treated parasites. The relative growth was higher for the Sir2rp3-WT parasites showing that Sir2rp3 induces resistance to both drugs, NFX and BZD, compared with WT and Sir2rp3-MUT cells (**Figures 2A, B**). More importantly, the resistance phenotype observed for Sir2rp3-WT cells was reverted when the assays were performed in the presence of salermide, another specific sirtuin inhibitor (**Figure 2**).

### Cytosolic and Mitochondrial ROS Levels Are Lower in Sir2rp3-WT Parasites After BZD and NFX Treatment

**Figure 3:**
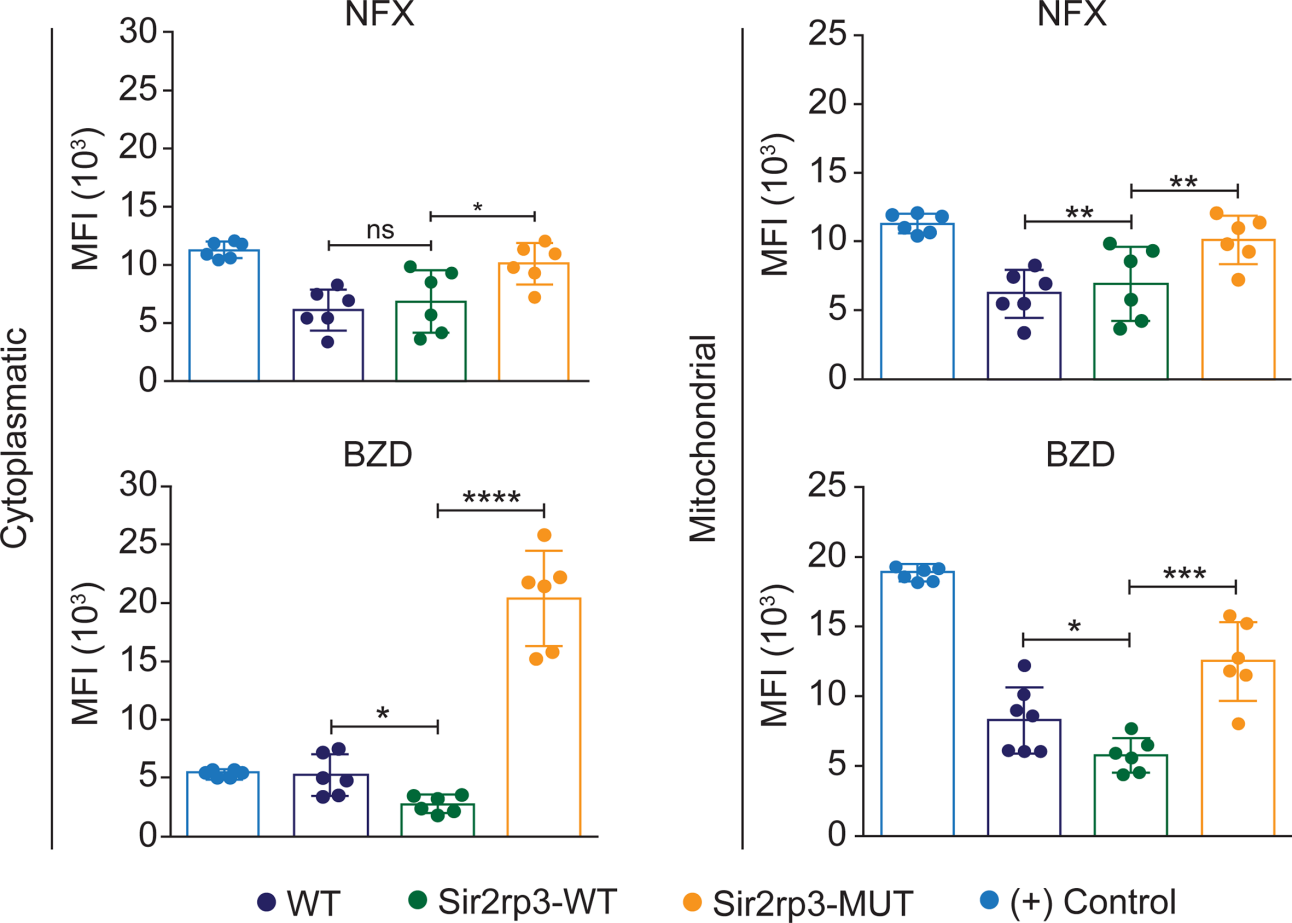
Mitochondrial sirtuin decreases ROS levels after BZD and NFX treatment. Sir2rp3-WT epimastigote presented lower cytosolic and mitochondrial ROS levels after BZD and NFX treatment compared with WT and Sir2rp3-MUT parasites. A significant increase of ROS levels was observed in Sir2rp3-MUT. WT parasites submitted to H_2_O_2_ were used as positive control (+). MFI, mean fluorescence intensity. All experiments were performed in triplicate. Statistical analyses were done using the Student’s *t*-test. BZD analysis ^*^ *p* = 0.0105; ^***^ *p* < 0.0003; ^****^ *p* < 0.0001. NFX analyses, ^*^ *p* = 0.0067; ^**^ *p* = 0.0019. ns, not significant.

To know if the BZD and NFX resistance observed in the Sir2rp3-WT parasites was related to reduction in the ROS levels, WT, Sir2rp3-WT, and Sir2rp3-MUT epimastigotes were cultivated in the presence of 25 μg/ml of BZD or 15 μM of NFX, corresponding to twice the EC_50_ of these drugs in the employed *T. cruzi* strain, for 24 h, and the levels of cytosolic and mitochondrial ROS were further quantified. Lower levels of ROS were detected in the Sir2rp3-WT cells compared with WT and Sir2rp3-MUT parasites (**Figure 3**), especially in the BZD-treated parasites. In contrast, the ROS levels observed for Sir2rp3-MUT cells were higher than the WT parasites, suggesting a deleterious effect caused by the overexpression of an inactive TcSir2rp3 form.

### TcSir2rp3 Overexpression Does Not Affect TcSODA Protein Expression

**Figure 4:**
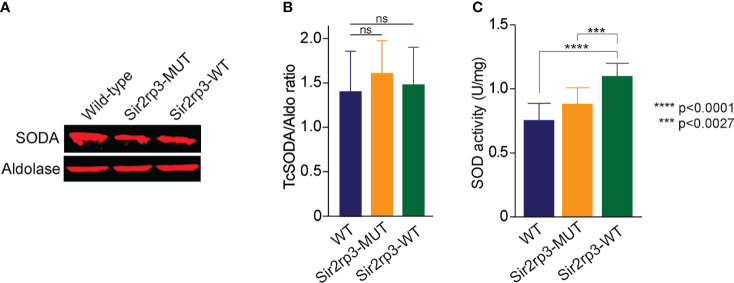
TcSir2rp3 overexpression increase superoxide dismutase activity in *T. cruzi*. **(A, B)** Comparative analyses of TcSODA protein expression in the wild-type, Sir2rp3-WT and Sir2rp3-MUT parasites. No significant differences were observed among the cell lines for all enzymes analyzed. Aldolase was used as loading control. TcSODA levels were determined for all cell lines from a triplicate experiment. **(C)** SOD activity of total extracts of parasites. The assays were carried out in triplicate. Statistical analyses were performed using the Student’s *t*-test. ^***^ *p* = 0.0027; ^****^ *p* < 0.0001. ns, not significant.

To investigate the mechanisms involved in the resistance to H_2_O_2_, menadione, BZD, and NFX, observed in the parasites overexpressing TcSir2rp3, we decided to compare the protein expression level of a key antioxidant enzyme, mitochondrial superoxide dismutase (TcSODA), among epimastigotes from WT, Sir2rp3-WT, and Sir2rp3-MUT. No large differences in protein expression levels were observed for TcSODA among the cell lines (**Figures 4A, B**).

### Superoxide Dismutase Activity Increases After TcSir2rp3 Overexpression

As human MnSOD activity was demonstrated to be negatively regulated by acetylation and that mitochondrial sirtuin SIRT3 is responsible to promote its deacetylation (Chen et al., 2011), we decided to measure superoxide dismutase activity in the parasites overexpressing TcSir2rp3.

Therefore, we measured the SOD activity in total protein extracts of WT, Sir2rp3-WT, and Sir2rp3-MUT epimastigotes. We observed an increase of approximately 40% in superoxide dismutase activity in Sir2rp3-WT protein extracts compared with those from WT and Sir2rp3-MUT (**Figure 4C**). This increase was not due to augmented protein expression as Western blot analyses using anti-TcSODA antibody showed similar levels of TcSODA among the parasites used in the activity assays (**Figures 4A, B**). This result suggests that TcSODA activity might be higher in Sir2rp3-WT parasites, and this could be associated to decreased levels of TcSODA acetylation promoted by sirtuin activity.

### TcSir2rp3 and TcSODA Interacts *In Vivo*

The set of experiments performed before suggests that TcSir2rp3 might be involved in oxidative stress response in *T. cruzi* by regulating the acetylation level of TcSODA. To gain insight in the regulatory function of TcSir2rp3 above TcSODA activity, we investigated the interaction of these two enzymes *in vivo* by protein immunoprecipitation assays.

**Figure 5:**
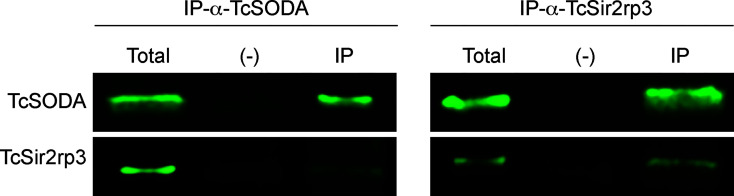
Interaction of TcSir2rp3 and TcSODA proteins. Immunoprecipitated samples with anti-TcSODA (left) or with anti-TcSir2rp3 (right) of total protein extracts of WT parasites were submitted to Western blot analyses with anti-TcSir2rp3 and anti-TcSODA. The two proteins, TcSir2rp3 and TcSODA, were detected in the immunoprecipitated fractions (IP). No signal was detected in the negative control samples (−) immunoprecipitated with anti-aldolase. Experiments were performed in triplicate, and the figure is a representative of the results obtained.

Total protein extracts from WT parasites were obtained and immunoprecipitated with anti-TcSODA and samples submitted to Western blot analyses with anti-TcSODA and anti-TcSir2rp3. As shown in **Figure 5**, TcSir2rp3 and TcSODA were detected in the immunoprecipitated fraction, indicating that these proteins interact with each other in the parasite. The negative control of the experiment was done through immunoprecipitation with an unrelated antibody, anti-aldolase, and no interaction was observed (**Figure 5**).

### K97 Acetylation Negative Regulates TcSODA *In Vitro* Activity

To further understand the function of lysine acetylation in the regulation of TcSODA activity, we submitted heterologous TcSODA in its native state (WT) to acetic anhydrite (AA) treatment, a chemical compound that acetylates any exposed lysine residues followed by TcSODA *in vitro* activity assays. Crescent concentrations of AA followed by Western blot with antiacetyl lysine antibody showed an increased in the acetylation levels of TcSODA heterologous protein (**Supplementary Figure S1A**). In parallel, AA treatment samples were used in the *in vitro* activity assays and we observed that acetylation by AA reduced TcSODA activity compared with nontreated samples (**Supplementary Figure S1B**). This result suggested that acetylation might negatively impact TcSODA enzyme activity.

**Figure 6:**
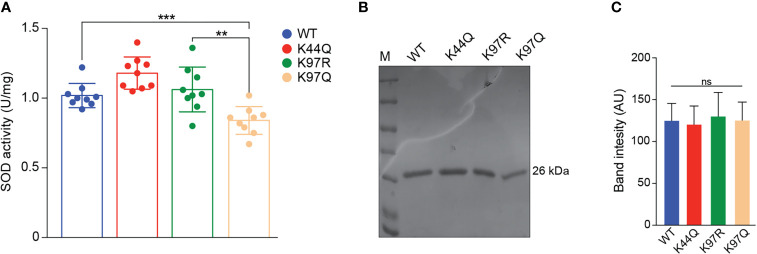
Superoxide dismutase activity of native and mutated versions of TcSODA. **(A)** Purified heterologous proteins of TcSODA (native and mutated versions) were submitted to superoxide dismutase assays using NBT oxidation method. A reduction of ~25% was observed for K97Q protein, which mimics acetylation, compared with the native (WT) or K97R versions. No significant difference was detected for K44Q compared with WT or K97R. All analyses were performed in triplicate. Statistical analyses were performed using the Student’s *t*-test. ^**^ *p* = 0.0028; ^***^ *p* = 0.0009. **(B)** Coomassie gel of TcSODA recombinant proteins was used in the *in vitro* activity assays. **(C)** Quantification of triplicate Coomassie gel of recombinant proteins used in the TcSODA activity assays. No significant differences were observed among the samples. ns, not significant.

In the *T. cruzi* acetylome, two lysine-acetylated sites were identified in TcSODA, K44 and K97 (Moretti et al., 2018). To evaluate the effect of acetylation at K44 and K97 residues in the regulation of enzyme activity, we used purified heterologous protein of WT and mutated versions mimicking acetylation at K44 and K97 (K44Q and K97Q) or nonacetylated state at K97 (K97R), to measure the superoxide activity *in vitro* by the NBT oxidation method. The acetylation of K97 reduced TcSODA activity in about 25% compared with WT protein (**Figure 6A**), which was not observed in the K97R that mimics nonacetylated state or K44Q that is located in a region distant from the tunnel of the catalytic site. The differences in the *in vitro* activity observed was not due to different protein concentrations used in the assays as demonstrated in **Figures 6B, C**.

### MD Simulations Suggests That the K97 Acetylation Affects the Salt-Bridge Pattern of TcSODA

**Figure 7:**
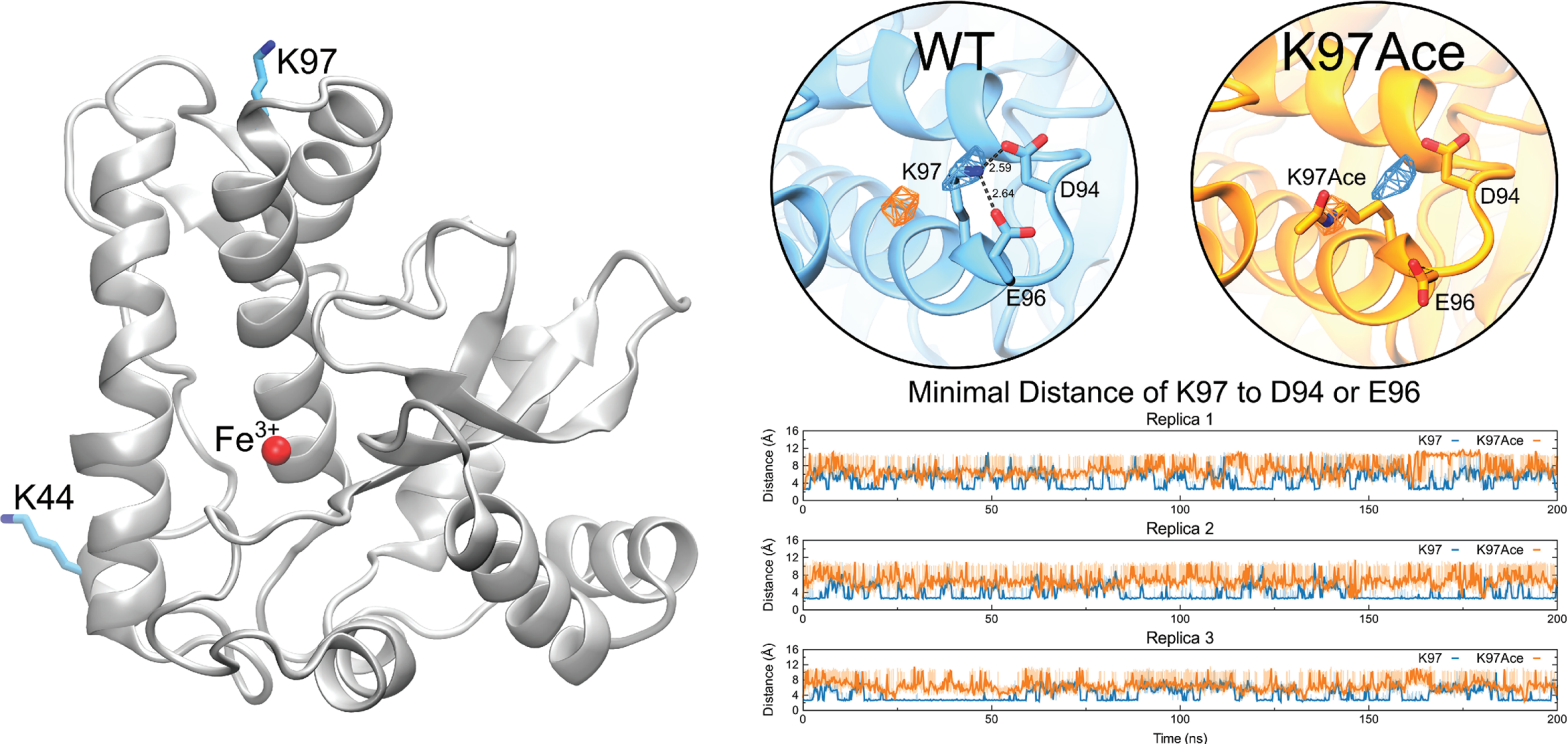
MD simulations reveal that K97 acetylation affects the salt-bridge pattern of TcSODA. Left, TcSODA structure as cartoon, highlighting the position of K97 residue and Fe^3+^, shown as cyan sticks and as a red sphere, respectively. Right, the orientation of K97 with respect to D94 and E96 in native (WT, in cyan) and acetylated (K97Ace, in orange) TcSODA, measured by the occupancy of the sidechain nitrogen atom of K97, showing that K97 is kept near these acidic residues whereas acetylation repeals it. This spatial change of K97 alters the salt-bridge formation between such residues, as shown by minimal distance measurements in all replicas.

To better understand how the acetylation pattern of TcSODA affects its ability to process superoxide, we performed MD simulations of the native (WT) and K97-acetylated (K97Ace) versions of TcSODA. The lysine 97 is placed at around 21 Å away from the catalytic site (**Figure 7**, left), and despite this, acetylation reduces the enzymatic activity of TcSODA by ~25% *in vitro*. Initially, we analyzed the conformational changes locally around the residue in both WT and mutant and detected that the sidechain nitrogen of TcSODA showed an evident difference in spatial occupancy comparing WT and K97Ace. The WT maintained the lysine crystallographic orientation, while the acetylated one presented its sidechain with a tendency to move away from that position (**Figure 7**, right). This behavior can be explained by the interaction with neighbor residues, in which K97 is able to interact to with two near-acidic residues, named D94 and E96, while K97Ace tends to be repealed due to charge modification caused by the acetyl group.

To compare the dynamical behavior of K97 in WT or acetylated with respect to neighbor acidic residues, the minimal distance between the sidechain nitrogen of K97 to the sidechain oxygen atoms of D94 or E96 was calculated (**Figure 7**, right). It clearly showed how the WT lysine orients towards both residues, showing interactions at distances bellow 3.5 Å, which correspond to salt-bridge interactions, in 52.21% of the time, while the acetylated enzyme showed such interaction in only 1.46%. This shows that the acetylation caused a ~36-fold reduction of salt bridges between K97 and D94 or E96 in TcSODA.

## Discussion

*T. cruzi* is exposed to different redox environments during its life cycle, and adaptation to these conditions determines the success of the infection (Perez-Molina and Molina, 2018; Lidani et al., 2019). To deal with this situation, the parasite has developed a sophisticated arsenal of antioxidant enzymes that help maintain redox homeostasis (Beltrame-Botelho et al., 2016). In this work, we showed for the first time that protein acetylation can regulate the oxidative stress response through the action of the sirtuin TcSir2rp3, a lysine deacetylase enzyme located in the mitochondria (Moretti et al., 2015). Here, we demonstrated that TcSir2rp3 deacetylation activity increased the resistance to different oxidants and reduced the levels of cytoplasmic and mitochondrial ROS. We show that the mitochondrial SOD of the parasite is one of the targets of the sirtuin, as the specific activity of the enzyme is increased upon acetylation both *in vitro* and *in vivo*. Based on molecular modeling, we suggest that acetylation of specific lysine residues negatively regulates TcSODA activity by affecting protein conformation, which causes changes in the active site.

Sirtuins are present from bacteria to humans and act by regulating diverse cellular processes from different cellular compartments (Choi and Mostoslavsky, 2014). SIRT3 is the main mitochondrial sirtuin in humans playing an essential role on the homeostasis of this organelle through the deacetylation of metabolic proteins, including those involved with ROS detoxification processes (Cooper and Spelbrink, 2008; Qiu et al., 2010). Also, it is known that SIRT3 knockout cells show high levels of ROS compared with wild-type cells (Ahn et al., 2008; Tao et al., 2010). Our results demonstrating that parasites overexpressing the mitochondrial sirtuin, TcSir2rp3, increased parasite resistance to different oxidant compounds (H_2_O_2_, menadione, NFX, and BZD) and reduced the levels of cytoplasmic and mitochondrial ROS are in agreement with what was observed in mammals, suggesting an evolutionary conservation of the mechanisms involved in the regulation of oxidative stress response.

The deleterious effect observed in the TcSir2rp3-MUT parasites for menadione and BZD resistance and ROS production might be a dominant negative effect caused by the overexpression of mutated enzyme that will not only compete for the target proteins but also for the NAD+ cofactor, with the endogenous protein. We observed the same scenario in the past during intracellular amastigote multiplication experiments (Moretti et al., 2015).

**Figure 8:**
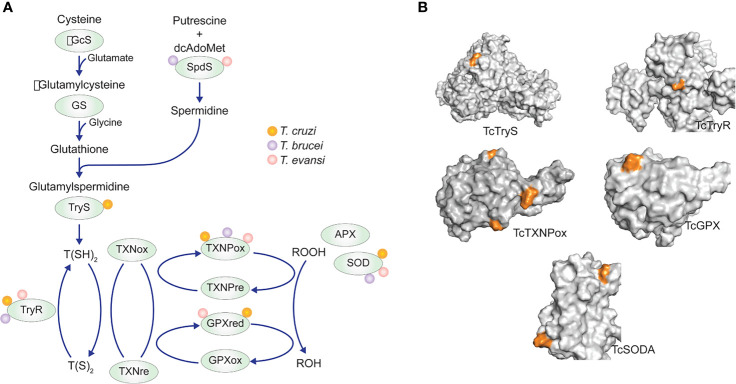
Antioxidant enzyme acetylation detected in *T. cruzi*, *T. brucei*, and *T. evansi* acetylomes. **(A)** Enzymes belonging to the tree main antioxidant pathways of trypanosomatids with one or more acetylation lysine sites. **(B)** Distribution of lysine-acetylated sites (orange) in the 3D protein structure of the five *T. cruzi* antioxidant enzymes detected in the acetylome of the parasite.

Although our data only provide evidence that TcSODA is regulated by acetylation, we cannot exclude that the resistance to oxidants depends on the levels of other antioxidant enzymes by different mechanisms. Nevertheless, five *T. cruzi* antioxidant enzymes were identified to be acetylated at different sites (**Figure 8** and **Supplementary Table S2**), suggesting that acetylation might affect their activities as observed for antioxidant enzymes from other organisms (Narita et al., 2019). The regulatory function of lysine acetylation has been demonstrated for different antioxidant enzymes in humans, such as peroxiredoxins (PrxI and PrxII), superoxide dismutase 1 (SOD1), and mitochondrial MnSOD (Parmigiani et al., 2008; Chen et al., 2011; Lin et al., 2015).

The SODs promote the dismutation of two-molecule superoxide $\left({\text{O}}_{2}^{-}\right)$ generating H_2_O_2_ and O_2_, thus participating in the first line of defense of cells against oxidative stress (Wang et al., 2018). Our findings showing that overexpressing the native form of TcSir2rp3 increased superoxide activity is similar with what was found for human SIRT3, where the reduced acetylation status of MnSOD increased the enzymatic activity, contributing to the protection of cells against oxidative stress (Tao et al., 2010; Chen et al., 2011). Moreover, the direct interaction of TcSir2rp3 with TcSODA in the epimastigote forms, as observed in the protein immunoprecipitation assays, supports the notion that TcSODA activity could be directly modulated by TcSir2rp3. It will be interesting to demonstrate the TcSODA-specific activity and determine the acetylation levels of the enzyme in the parasites overexpressing TcSir2rp3.

A comparison of the TcSODA and human MnSOD structures shows that the relative position of the acetylated K97 residue in *T. cruzi* is similar to that of the K68 residue in human MnSOD (Phan et al., 2015), suggesting that acetylation at K97 could regulate TcSODA activity. Through site-directed mutations, we demonstrated that the replacement of K97 residue, the corresponding residue to K68 in humans, of TcSODA by glutamine, which mimics an acetylated lysine, causes decreased activity of the protein *in vitro*, as for human MnSOD (Chen et al., 2011; Lu et al., 2015). This effect was not seen when we replaced K97 with arginine, which mimics a deacetylated lysine, or when we perform a mutation in position K44, located in a region distant from K97. Thus, these data further support the idea that TcSODA is regulated by acetylation. Moreover, TcSODA K97 residue is conserved in *T. brucei* and *T. evansi*, corresponding to K101, which was detected to be acetylated and is located at a similar region in the protein 3D structure (**Supplementary Figure S2**; **Supplementary Table S2**), indicating a conserved function in other trypanosomatids.

The direct effect of acetylation on the regulation of MnSOD activity has been proposed more than 30 years ago, and it was hypothesized that the tetrameric monomer complex of MnSOD would create a positively charged funnel structure that would facilitate the attraction of negatively charged ${\text{O}}_{2}^{-}$ molecules to the catalytic site of MnSOD, and that the acetylation of lysines positioned at the funnel would cause electrostatic changes to the surface, thus affecting the activity of the enzyme (Benovic et al., 1983; Fridovich, 1983). Interestingly, we observed *in silico* that K97 acetylation can induce particular conformational changes in TcSODA with respect to hydrogen bonding pattern to neighbor residues, specifically D94 and E96, suggesting a key participation of this residue to modulate the affinity to ${\text{O}}_{2}^{-}$ by changing the charge availability on the surface of the enzyme. These findings suggest that lysine residues play a central role in TcSODA activity by sequestering negative charges of D94 and E96 in the native form and exposing such negatively charged residues to the solvent when acetylated, modulating the affinity of the enzyme to ${\text{O}}_{2}^{-}$ .

In addition, several studies demonstrate that replacement of MnSOD K68, which is located in a ring of 11 positively charged residues surrounding the active site and forming the characteristic funnel structure of the enzyme (Borgstahl et al., 1992), by a residue mimicking an acetylated lysine or by an acetylated lysine, negatively affects the activity of the enzyme, and that this effect has a direct relationship with changes in the electrostatic potential of the protein, causing an increase in negative charges in the funnel region of the protein and decreasing the interaction of ${\text{O}}_{2}^{-}$ with the catalytic site (Chen et al., 2011; Lu et al., 2015; Lu et al., 2017). Indeed, lysine acetylation modulates the ${\text{O}}_{2}^{-}$ affinity by changing the global charge of the enzyme. In the present work, we showed that lysine residues can be important to modulate the charge availability of TcSODA by sequestering negatively charged residues near the catalytic site by salt-bridge formation, suggesting that the molecular mechanism in free radical processing is likely more sophisticated than expected.

In conclusion, our results demonstrated for the first time the impact that lysine acetylation has in the regulation of redox state in trypanosomatids, contributing to better understand the mechanisms employed by *T. cruzi* to deal with the oxidant environmental during the progression of the infection. These observations open the opportunity to explore protein acetylation as a potential drug target in this parasite, possibly through combinatory therapy with the existent drugs. Moreover, this work helps to expand our knowledge about the function of lysine acetylation in the regulation of cellular processes in eukaryote organisms.

## Data Availability Statement

The original contributions presented in the study are included in the article/**Supplementary Material**. Further inquiries can be directed to the corresponding author.

## Author Contributions

Conceptualization: LS, VS, AG, AS, MF, SS, and NSM. Methodology: LS, AG, and NSM. Validation: LS, AG, and NSM. Investigation: LS, VS, AG, AS, and NSM. Resources: SS, MF, and NSM. Writing—original draft preparation: LS, AG, AS, SS, and NSM. Writing—review and editing: LS, VS, AG, AS, MF, SS, and NSM. Visualization: AG, SS, and NSM. Supervision: NSM. Project administration, NSM. Funding acquisition: SS and NSM. All authors have read and agreed to the published version of the manuscript.

## Funding

This work was supported by the Fundação de Amparo à Pesquisa do Estado de São Paulo (FAPESP), grants 2018/09948-0 and 12/09403-8 to NSM, 15/22031-0 and 2020/07870-4 to NSM and SS, and 14/03714-7 to VS; Conselho Nacional de Desenvolvimento Científico e Tecnológico (CNPq), grants 424729/2018-0 to NSM and 302883/2017-7 to MF; Instituto Nacional de Ciência e Tecnologia de Vacinas (INCTV) and scholarship 302972/2019-6 to SS and scholarship 165639/2017-2 to LS; and Coordenação de Aperfeiçoamento de Pessoal de Ní-vel Superior scholarship 88887.506120/2020-00 to AS.

## Conflict of Interest

The authors declare that the research was conducted in the absence of any commercial or financial relationships that could be construed as a potential conflict of interest.

## Publisher’s Note

All claims expressed in this article are solely those of the authors and do not necessarily represent those of their affiliated organizations, or those of the publisher, the editors and the reviewers. Any product that may be evaluated in this article, or claim that may be made by its manufacturer, is not guaranteed or endorsed by the publisher.
