# Supplementary material for: Mitochondrial Sirtuin TcSir2rp3 Affects TcSODA Activity and Oxidative Stress Response in Trypanosoma cruzi
Source: Front Cell Infect Microbiol. 2021 Nov 11;11:773410. doi: 10.3389/fcimb.2021.773410 (PMC8632061; doi:10.3389/fcimb.2021.773410)
Supplement: Supplementary file 1 [file DataSheet_1.pdf]

Supplementary material

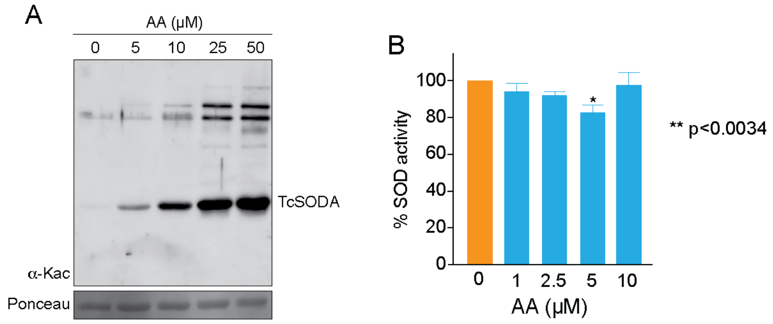

Sup. Figure 1. Effect of AA in the TcSODA in vitro enzyme activity. A. Treatment of TcSODA with AA increases protein acetylation. TcSODA-WT purified heterologous protein was submitted to treatment with different concentrations of AA and samples used in western blot analyses with anti-acetyl-lysine antibodies. The levels of lysine acetylation increased proportionally with AA concentrations. **B.** Treatment of TcSODA-WT with different concentrations of AA decreased the enzymatic activity *in vitro* compared to non-treated proteins. All the experiments were performed in triplicate.

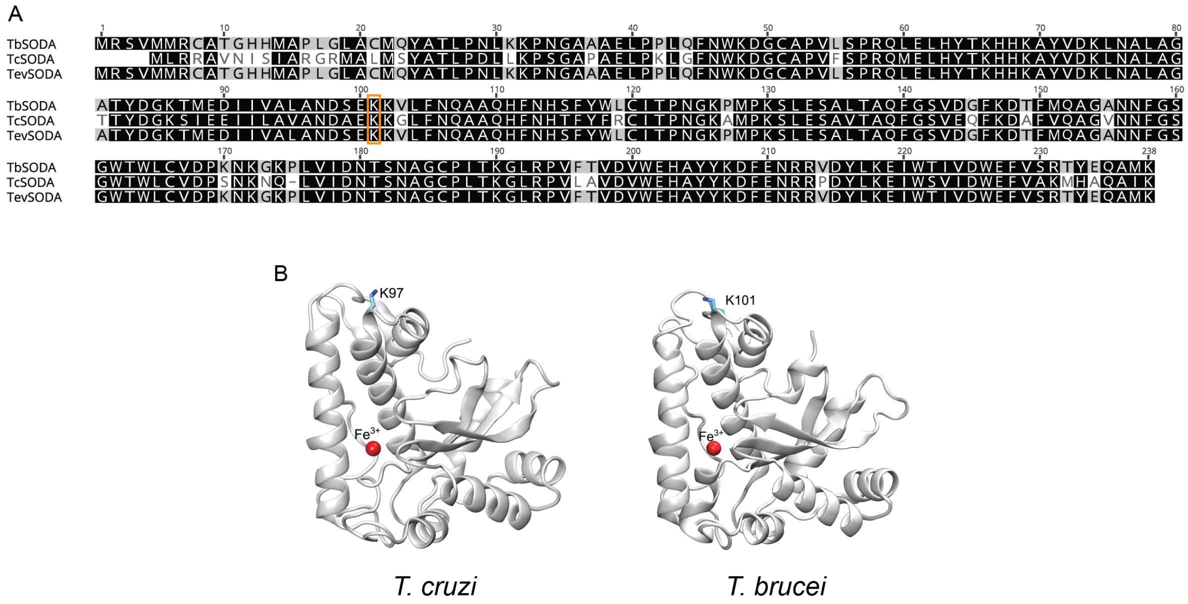

Sup. Figure 2. Conservation of K97 residue among *T. cruzi*, *T. brucei* and *T. evansi*. A. Amino acid sequence alignment of TcSODA (TcCLB.509775.40) and TbSODA (Tb927.5.3350) highlighting the conservation of lysine residue K97 between the two parasites. The conserved lysine residue is highlighted in orange and corresponds to K101 in *T. brucei* and *T. evansi*. **B.** Comparative 3D protein structure of TcSODA (PDB 4H3E) and TbSODA demonstrating that K101 is located at similar region of K97, suggesting a conserved regulatory mechanism in both parasites. *T. brucei* 3D protein structure was predicted using SwissModel https://swissmodel.expasy.org). *T. evansi* 3D structure was not included due to the high similarity with TbSODA protein.

Supplementary tables

Table S1. Oligonucleotides used to generate heterologous TcSODA

Name	Sequence
TcSODA-WT (F)	5' TCTAGACATATGTTGAGACGTGCGGTGAA 3'
TcSODA-WT (R)	5' CCGGATCCTTATTTTATGCCTGCGCATGCAT 3'
TcSODA-K44Q	5' AGTTGGGGTTTAACTGGCAGGATGGA 3'
TcSODA-K97R	5' GTCGCAAATGACGCCGAGAGGAAGGG 3'
TcSODA-K97Q	5' GTCGCAAATGACGCCGAGCAGAAGGG 3'

* Underlined nucleotides represent the mutations inserted in the TcSODA sequence.

Table S2. Acetylated AD enzymes detected in *T. cruzi*, *T. brucei* and *T. evansi* acetylomes

T. cruzi		
TritrypDB ID	Gene name	Kac sites detected
TcCLB.509099.50	Trypanothione synthetase	604
TcCLB.484299.10	Trypanothione reductase	147
TcCLB.509775.40	iron superoxide dismutase A, mitochondrial	44; 97
TcCLB.503899.130	Glutathione peroxidase	98
TcCLB.487507.10	Tryparedoxin peroxidase	7; 64; 120; 168

T. brucei		
TritrypDB ID	Gene name	Kac sites detected
Tb927.11.15910	iron superoxide dismutase	22; 31; 138
Tb927.11.15820	iron superoxide dismutase C, mitochondrial	75
Tb927.11.15020	iron superoxide dismutase	39
Tb927.5.3350	iron superoxide dismutase A, mitochondrial	66; 74; 101
Tb927.7.1130	trypanothione/tryparedoxin dependent peroxidase 2	43; 55; 89; 91; 115
Tb927.8.1990	peroxidoxin	56; 64; 139; 165; 197; 219; 224
Tb927.10.10390	trypanothione reductase	99; 144; 388
Tb927.9.7770	spermidine synthase	220; 222; 273; 281
Tb927.9.5860	tryparedoxin peroxidase	27; 28; 108; 120; 161; 168
Tb927.7.4000	glutathione synthetase	223

T. evansi		
TritrypDB ID	Gene name	Kac sites detected
TEV003095.1	iron superoxide dismutase, putative	22; 31; 66; 74; 101; 135
TEV006051.1	glutathione peroxidase-like protein 3, putative	43; 55; 57; 89; 91; 115
TEV000603.1	Chain A, Trypanothione Reductase	60; 99; 144; 266
TEV001721.1	spermidine synthase, putative	38; 222; 273; 281
TEV004742.1	tryparedoxin peroxidase	64; 130; 139; 165; 197; 219; 224
TEV006005.1	tryparedoxin peroxidase	27; 28; 120; 161; 168
TEV006051.1	glutathione peroxidase-like protein 3, putative	43; 55; 57; 89; 91; 115
